# An Electroanalytical Flexible Biosensor Based on Reduced Graphene Oxide-DNA Hybrids for the Early Detection of Human Papillomavirus-16

**DOI:** 10.3390/diagnostics12092087

**Published:** 2022-08-28

**Authors:** Reema Rawat, Souradeep Roy, Tapas Goswami, Ashish Mathur

**Affiliations:** 1 Department of Allied Sciences, School of Health Sciences and Technology, University of Petroleum and Energy Studies, Dehradun 248007, India; 2 Centre for Interdisciplinary Research and Innovation (CIDRI), University of Petroleum and Energy Studies, Dehradun 248007, India; 3 Department of Chemistry, School of Engineering, University of Petroleum and Energy Studies, Dehradun 248007, India; 4 Department of Physics, School of Engineering, University of Petroleum and Energy Studies, Dehradun 248007, India

**Keywords:** HPV-16, cervical cancer, rGO/DNA hybrids, flexible genosensor, screen-printed electrodes, point-of-care

## Abstract

Human Papilloma Virus 16 (HPV 16) is the well-known causative species responsible for triggering cervical cancer. When left undiagnosed and untreated, this disease leads to life-threatening events among the female populace, especially in developing nations where healthcare resources are already being stretched to their limits. Considering various drawbacks of conventional techniques for diagnosing this highly malignant cancer, it becomes imperative to develop miniaturized biosensing platforms which can aid in early detection of cervical cancer for enhanced patient outcomes. The current study reports on the development of an electrochemical biosensor based on reduced graphene oxide (rGO)/DNA hybrid modified flexible carbon screen-printed electrode (CSPE) for the detection of HPV 16. The carbon-coated SPEs were initially coated with rGO followed by probe DNA (PDNA) immobilization. The nanostructure characterization was performed using UV-Vis spectroscopy, Fourier transform infrared (FTIR) spectroscopy, Raman spectroscopy and X-ray diffraction (XRD) techniques. Cyclic voltammetry (CV) and electrochemical impedance spectroscopy (EIS) were employed to study the electrochemical characterization of the nano-biohybrid sensor surface. The optimization studies and analytical performance were assessed using differential pulse voltammetry (DPV), eventually exhibiting a limit of detection (LoD) ~2 pM. The developed sensor was found to be selective solely to HPV 16 target DNA and exhibited a shelf life of 1 month. The performance of the developed flexible sensor further exhibited a promising response in spiked serum samples, which validates its application in future point-of-care scenarios.

## 1. Introduction

Cancerous infections arising due to the incursion of Human Papillomavirus (HPV) in the cervix leads to increasing fatalities among the women of developing nations, which results in devastating outcomes [1]. The annual global cases related to cervical cancer has been reported to be as high as 5.2 Lakhs to which India contributes approximately 1.2 Lakh cases every year [2]. HPVs are often classified into two types—high-risk and low-risk, with the former category including HPV 16 and HPV 18 types. These species are equally fatal as evidenced by their prevalence in more than half (70%) of cervical cancer scenarios [2]. In numerous cases of untimely diagnoses, such cancers become malignant which leads to fatal outcomes such as lymph node infringement, inflammation and eventually death [3]. Therefore, early detection of cervical cancer by quantifying the presence of HPV-16 is of utmost importance in order to avoid life-threatening circumstances. 

The conventional strategies for detecting the presence of HPV-16 infections includes histological measurements and analysing cytological fluctuations using biopsy or Papanicolaou (Pap) screening methods [4]. The strategies involving the latter, hybrid capture assay and colposcopy, suffer from low sensitivity and specificity while being time consuming [5]. Meanwhile, polymerase chain reaction (PCR) has comparatively high sensitivity and is currently being widely employed for HPV detection, albeit with limitations of its own, such as the requirement of skilled manpower, high cost per test and tedious DNA extraction protocol [5]. Furthermore, the presence of limited cyto technicians in every hospital or clinic in developing nations has detrimental effects on the affected patients, leading to delayed treatment with dreadful outcomes [6]. It has also been observed that some interpretations of the cytology images are often vague, while being greatly prone to interference in the presence of blood and vaginal discharge leading to false positive or negative results [6]. It must be noted that the above screening tests are undertaken specifically after observing cervical cancer-related symptoms, whereby the situation may have worsened by the time patients begin with implementing the necessary treatment protocols [5,6]. Therefore, it becomes extremely important to develop highly sensitive, rapid and cost-effective biosensing platforms which can detect the occurrence of cervical cancer, by monitoring HPV 16 activity, at its onset. 

Electrochemical transduction has usually been in use in biosensor design because of its high degree of sensitivity, LoD and selectivity within a wide detection range [7,8,9,10,11]. Such transduction strategies are extremely suitable for integration with flexible electrodes, and as such, have proved immensely beneficial in the point-of-care scenario [12]. The IDTechEx has estimated a $8 billion rise in the printed and flexible electrochemical sensors market by 2025 [13]. The implementation of flexible electrodes offers numerous advantages such as low cost, high stretchability, hassle-free handling and disposability thereby contributing to minimizing e-waste (electronic waste). Integration of such flexible sensing electrodes with suitable nanomaterials further adds to electroanalytical properties. The high surface-to-volume ratio providing enhanced receptor immobilization and quantum confinement features resulting in superior electronic conductivity or signal amplification have proved to be a boon in the development of electrochemical biosensors [14,15,16,17]. Graphene-based nanomaterials have been deployed in a variety of applications due to diverse possibilities of functionalization, biocompatibility and appreciable stability under physiological conditions, henceforth making them versatile nano-transduction electrode modifiers [18,19,20,21]. Reduced graphene oxide (rGO) is a 2D material which exhibits unusual physical and chemical properties, allowing us to exploit the high electronic conductivity of exposed graphene edge planes as well as anchoring various biomolecules via the amide linkage—the latter using the oxygen functionalities present on rGO sheets. Furthermore, due to high surface-to-volume ratio and rich π conjugated features, the rGO nanostructures offer an excellent electrode surface for the efficient immobilization of bio-receptors, for enhanced detection processes [21]. 

In the present study, we have employed rGO/DNA nano-biohybrid-coated carbon screen-printed flexible electrodes for the electroanalytical genosensing of cervical cancer as a function of HPV-16 concentrations. The rGO sheet acts as a stable support which is expected to bind probe DNA (PDNA) strands using the established carbodiimide coupling, thereby resulting in a cost-effective, robust and sensitive DNA-based sensor for early monitoring of cervical cancer.

## 2. Experimental

### 2.1. Materials and Reagents 

The carbon-coated screen-printed flexible electrodes (φ = 3 mm) were purchased from Micrux Technologies, Asturias, Spain. Graphite powder, potassium permanganate (KMnO_4_), Methylene Blue (MB), Dimethyl Formamide (DMF), sodium dibasic and monobasic salts (Na_2_HPO_4_ and NaH_2_PO_4_) were purchased from Fisher Scientific, Mumbai, India. N-Hydroxy Succinimide (NHS), hydrazine hydrate and 1-Ethyl-3-(3-dimethylaminopropyl) Carbodiimide (EDC) were purchased from Merck, Mumbai, India. The amine-modified probe, target and complimentary DNA strands were purchased from GeneBio Solutions, Dehradun, India, with the sequences as per previous reports [6]. The DNA (probe and target) aliquots were made in (Tris-EDTA) TE buffer and stored at 4 °C. In all the experiments de-ionized (DI) water (18.2 MΩ) was used.

### 2.2. Synthesis of Reduced Graphene Oxide (rGO) Nanostructures

The synthesis of rGO nanostructure from graphite powder was carried out using modified Hummer’s method and described elsewhere [22,23]. A batch of 2 g of graphite powder (~325 mesh) was initially dispersed in 100 mL concentrated sulfuric acid. Then, KMNO_4_ powder (~6 g) was gradually added to the mixture maintaining the temperature at ~5 °C in an ice bath under vigorous stirring. After the complete addition of KMnO_4_, 150 mL of distilled water was added and kept under stirring for 1 h. Then the mixture was heated at 60 °C for 1 h. Then 20 mL of 30% H_2_O_2_ (Hydrogen Peroxide) solution was added to terminate the oxidation reaction by reducing the residual KMnO_4_. The brown colour suspension was centrifuged at 6000 rpm and washed several times with double distilled water to get to a neutral pH. The final brown colour solid was dried in a vacuum oven for 24 h. The dry graphene oxide was then dispersed in 100 mL N-methyl-2-pyrolidone. To this dispersion, 2 mL of hydrazine hydrate was added and kept under heating at 60 °C for 30 min to yield a black coloured suspension, which was centrifuged and washed with distilled water to obtain the precipitate of reduced graphene oxide.

### 2.3. Characterization of rGO Nanostructures

The UV-visible absorption spectrum of the dispersion of the rGO nanostructure in N-methyl-2-pyrolidone was collected with an UV-visible spectrophotometer (Lamda 35, Perkin Elmer, New Delhi, India). The powder X-ray diffractogram was used to obtain the phase structure of the nanostructure using a D8 Advance Eco- Bruker X-ray diffractometer. The FTIR spectra (400–4000 cm^−1^) were measured using a spectrometer (Perkin Elmer, New Delhi, India) to characterize the functional groups present in the sample. To characterize the vibrational band present in rGO nanosheets, Raman spectrum was recorded using a Raman Spectrometer (Research India, Bhopal, India).

### 2.4. Sensor Surface Fabrication

The working region (φ = 3 mm) of carbon screen-printed flexible electrode was initially modified with a 2 μL of 1 mg/mL rGO and was left for drying at 25 °C for 2 h. The nano-enabled carbon working electrode was further coated by immobilizing 2 μL of HPV-16 PDNA via the EDC-NHS coupling, and was allowed to stabilize for 24 h at 4 °C, which resulted in the development of an rGO/PDNA-based HPV-16 biosensor for the detection of HPV-16. A similar method of DNA-based sensor fabrication was followed in a previous study [24]. 

### 2.5. Electrochemical Analyses

The electrochemical studies were conducted in a three-electrode configuration, in DropSenseuStat i-400 potentiostat, in which an rGO/PDNA-coated carbon electrode served as the working electrode, while separate Ag/AgCl and carbon pads served as reference and counter electrodes, respectively. The electrolyte used is the Phosphate Buffer Saline—Methylene Blue redox indicator (PBS-MB, 0.1 M/1 µM pH 7.4). The preliminary electrochemical characterizations of CSPE/rGO/PDNA electrodes were performed using CV within −0.7 to 0.7 V at 100 mV/s, and EIS within 0.1 Hz–0.1 MHz at 100 mV. The sensor optimization with respect to PDNA concentrations (10–40 µM), incubation time (1–20 mins), electrolyte pH (6.2–7.4) and effect of temperature (10–50 °C) on the HPV-16 recognition process were performed using differential pulse voltammetry (DPV) within −0.6 V to 0.4 V at 100 mV/s. Under optimized conditions, the latter technique was further deployed in analytical sensing studies in which HPV-16 concentration was varied from 1 pM–1 µM. The sample volume was fixed at 70 µL for each concentration scan.

## 3. Results and Discussion

### 3.1. Microstructural Surface Characterization

**Figure 1:**
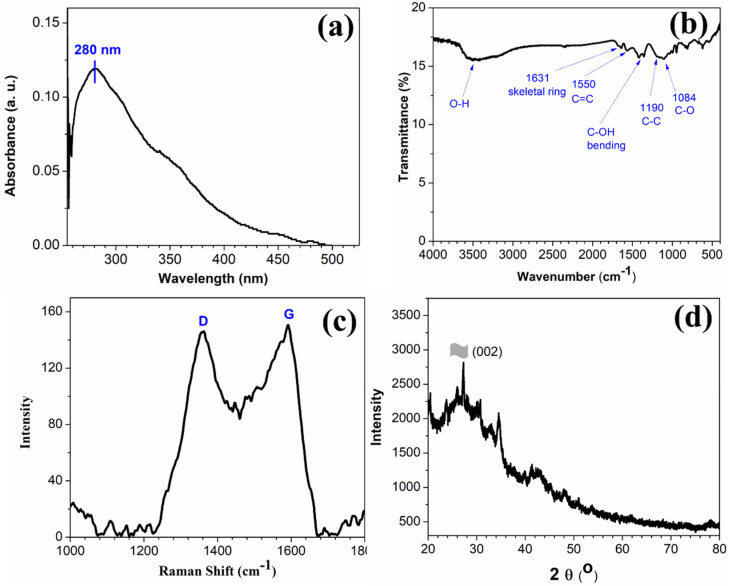
(**a**) UV-Vis spectrum of rGO dispersed in N-methyl-2-pyrolidone, (**b**) FTIR spectrum, (**c**) Raman spectrum, and (**d**) powder XRD pattern of as-prepared rGO.

The UV-Vis spectrum in Figure 1a of as-prepared rGO shows a peak centered at ~280 nm which is red-shifted in comparison to the pure GO [23].

This absorption band can be attributed to the π → π* transition of the C-C bonds, and the observed red-shifts indicate that, upon reduction of GO using hydrazine hydrate, the electronic conjugation is reduced as well as the structural ordering is enhanced [23]. The FTIR spectrum in Figure 1b of rGO exhibits the characteristic peaks of C-C conjugation and C–C band stretching vibrations at 1550 and 1190 cm^−1^, respectively. The absence of a vibrational band of C=O group (1734 cm^−1^) and the reduction in intensity of peaks at ~1228 cm^−1^ and 1084 cm^−1^ corresponding to oxygen functionalities confirm the conversion of GO to rGO in the presence of hydrazine hydrate [25,26]. The Raman spectrum (shown in Figure 1c) of the as-prepared rGO shows the characteristics D and G band. The D and G Raman bands represent the carbon sp^3^ vibrations in disordered graphene and the sp^2^ bonded in-plane vibration of carbon atoms, respectively. The calculated I_D_/I_G_ ratio of the as-synthesized rGO was found to be 0.97, which is higher than that of GO. This indicates a notable reduction in sp^3^-hybridsed carbon to sp^2^ bonded 2D carbon nanosheets [27]. The morphological structure of the rGO nanostructure was further confirmed using XRD analysis. The peak at 2θ value of 26° in the XRD spectra (Figure 1d) is attributed to the (002) crystal plane of the rGO sheets [28,29,30].

### 3.2. Response at Electrode Fabrication Stages

**Figure 2:**
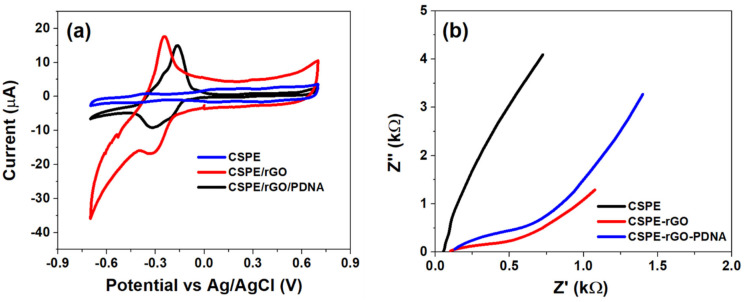
(**a**) CV plot within −0.7 to 0.7 V at 100 mV/s and (**b**) Nyquist plot recorded between 0.1 Hz–0.1 MHz (100 mV A.C) at different stages of sensor fabrication.

The initial electrochemical characterization of CSPE, CSPE/rGO and CSPE/rGO/PDNA surfaces was performed using CV and EIS techniques. The CV studies were performed within −0.7 V to 0.7 V at 100 mV/s, while EIS between 0.1 Hz–0.1 MHz (100 mV A.C). The resulting voltametric and Nyquist profiles are shown in Figure 2 below.

It can be observed from Figure 2a that the CSPE/rGO surface exhibits a comparatively higher peak anodic current response (I_pa_~18 µA) than that observed at the pristine CSPE surface (I_pa_~2 µA). The nine-fold increase in I_pa_ in the nano-modified electrode is primarily due to the high surface area and quantum confinement effects. The selection of MB as the redox indicator also has a major impact in enhancing the interface kinetics due to it being cationic in nature [31,32]. This results in a strong electrostatic attraction between MB and carboxyl groups of the rGO/PDNA surface leading to direct electron transfer to the electrode. However, the immobilization of HPV-16 PDNA decreases the overall interfacial electron transport due to the insulating nature of single-stranded DNA molecules, as evidenced by a decrease in I_pa_ to ~15 µA [33]. 

The Nyquist spectrum of Figure 2b indicates the presence of various interfacial phenomena corresponding to the bulk and electrode–electrolyte interfaces. Consequently, the CSPE/rGO/PDNA surface exhibits a diametrically smaller spectrum as indicated with a low charge transfer resistance. This is in contrast to that observed at the pristine CSPE electrode, which is attributed to the presence of a large surface area and the ballistic conduction of rGO nanostructures, facilitated by a strong electrostatic attraction between cationic MB and anionic oxygen species on the electrode surface. Meanwhile, the spectrum of the CSPE/rGO/PDNA electrode exhibits comparatively higher R_ct_ than the nanocomposite-modified surface due to the presence of a passivating DNA layer onto the electrode [34]. This situation is observed to be corroborated in the CV plots of Figure 2a. It can also be observed that the modified electrodes exhibit a diffusion mediated process, corresponding to the migration of MB towards the electrodes from the bulk electrolyte. The diffusion impedance appears to be higher on the PDNA-immobilized surface due to increased steric hindrance to the concentration-gradient-mediated flow of MB molecules towards the electrode. 

Therefore, the distinct response observed at each stage of sensor fabrication suggests successful fabrication of the CSPE/rGO/PDNA bioelectrode, which has been used in further analyses.

### 3.3. Optimization of CSPE/rGO/PDNA Based Genosensing Surface

**Figure 3:**
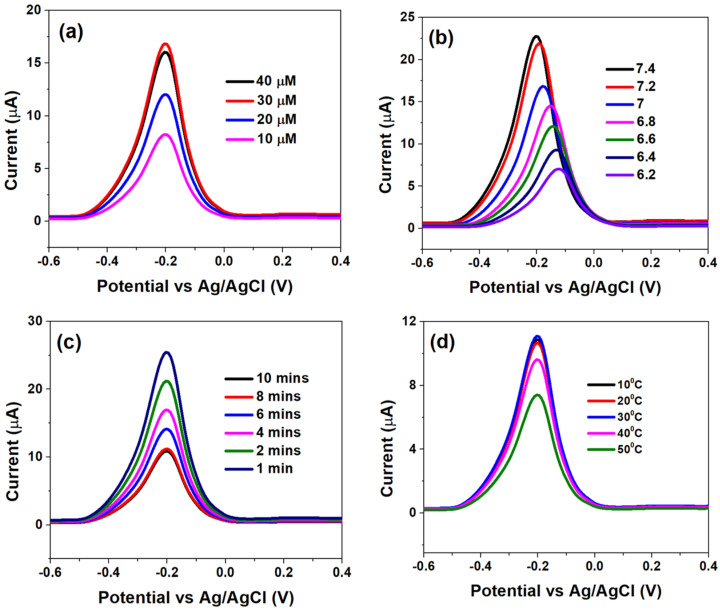
Differential pulse voltammograms obtained at (**a**) different HPV-16 PDNA concentrations, (**b**) various pH within 6.2–7.4, (**c**) incubation time of 1 μM HPV-16 TDNA at PBS-MB (0.1 M/1 μM, pH 7.4) and (**d**) effect of temperature variation on DNA hybridization, optimized at 10 min in PBS-MB (0.1 M/1 μM, pH 7.4).

The genosensor surface optimization analyses, with respect to PDNA concentrations, pH, incubation times and temperature variation, were assessed using DPV and are shown in Figure 3. 

Figure 3a indicates that the I_pa_ significantly increased when the concentration of PDNA was increased from 10 µM to 30 µM; which was primarily due to efficient binding of MB molecules with free guanine bases and consequent faster electron transfer. However, further increasing the PDNA concentration to 40 µM led to a subsequent decay in the electron transfer kinetics (decrease in I_pa_), which can be attributed to a high degree of steric hindrance offered by multiple adsorbed layers of PDNA, to the MB molecules. Therefore, an optimum PDNA concentration of 40 µM was chosen for further analyses.

Figure 3b indicates the effect of pH on the sensor interface kinetics. The alkaline environment is favourable for MB whereby the latter is reported to form cations at higher pH, in which case OH^−^ is adsorbed to the PDNA-modified bio-electrode surface to form negatively charged adsorption centres, which promotes the adsorption of MB ions [35,36]. This is indicated by high I_pa_ at pH 7.4 and its drastic reduction, followed by shifting of the voltammograms towards higher potentials, at lower pH which indicates slow electron transfer at the latter. Thus, pH 7.4 was chosen as the optimum for studying the analytical response to various HPV-16 TDNA concentrations. The effect of various incubation times on the hybridization process is shown in Figure 3c. A 50 μL volume of 1 μM TDNA was precisely dropped onto the PDNA-modified electrodes after which the hybridization time was varied from 1 to 10 min by employing PBS/MB (0.1 M/1 μM, pH 7.4). The current response was observed to decrease from ~25 µA to ~10 µA upon increasing the hybridization time from 1 to 8 min; which is an indication of efficient hybridization of the two single-stranded DNA into a double-helix structure [29]. However, the I_pa_ became saturated at 10 min indicating complete hybridization of PDNA and TDNA strands. Furthermore, in order to assess the heat stability of the genosensor upon hybridization of the DNA strands, the temperature was varied from 10 °C to 50 °C. 

It can be observed from Figure 3d that I_pa_ insignificantly differed as temperature was increased from 10 °C to 30 °C, while the former drastically increased at 40 °C and beyond, indicating DNA denaturation. Therefore, the optimum temperature range for the operation of genosensor was defined at 10 °C to 30 °C. 

### 3.4. HPV-16 Detection at CSPE/rGO/PDNA Electrodes

**Figure 4:**
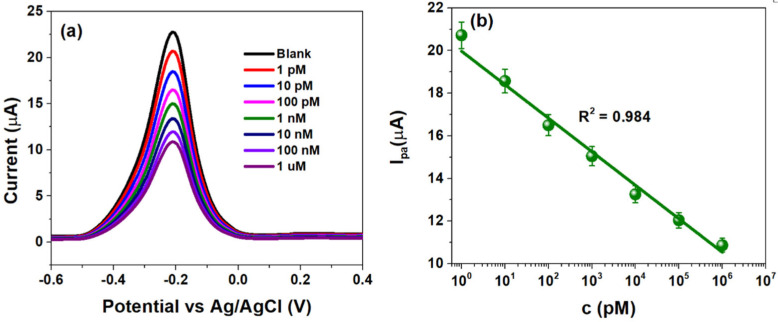
(**a**) Differential pulse voltammograms obtained at HPV-16 TDNA within 1 pM–1 µM and (**b**) sensor calibration obtained at −0.22 V (−220 mV) within the mentioned concentration range.

The analytical performance of CSPE/rGO/PDNA electrodes was studied using DPV within −0.6 to 0.4 V (100 mV/s). The HPV-16 TDNA concentration was varied from 1 pM–1 µM, and the corresponding plots are highlighted in Figure 4a.

The voltammograms indicate that a decrease in peak anodic currents (I_pa_) upon successive additions of HPV-16 TDNA as evidenced by an approximately two-fold decrement at 1 µM TDNA from the response of 1 pM TDNA as shown in Figure 4a. The I_pa_ was calculated to be ~20.72 µA at a concentration of 1 pM, which decreased to ~10.83 µA at 1 µM due to the formation of the bulky double-helix structure upon probe and target DNA hybridization [37]. The I_pa_ at various TDNA concentrations are observed at −220 mV (−0.22 V), at which the developed CSPE/rGO/PDNA sensor was calibrated. Figure 4b indicates the calibration plot demonstrating linearity between I_pa_ and TDNA concentrations (1 pM–1 µM), with line equation log{I_pa_ (µA)} = −1.57 log {c(µM)} + 19.97 (R^2^ = 0.99). In such a scenario, sensor currents of ~21 µA and ~11 µA indicate the absence and presence of HPV-16 infection, respectively. The LoD of the flexible HPV-16 sensor was calculated to be ~2 pM using the well-established 3σ rule [38]. Table 1 highlights a comparison of few parameters of the CSPE/rGO/PDNA platform with a few reported ones.

It can be observed from the above table that the developed rGO/PDNA-based sensor exhibits picomolar-level LoD within a wide HPV-16 concentration range (1 pM–1 μM), as compared to few reported ones. Such enhancement can be attributed to efficient immobilization of PDNA onto the rGO electrodes via the strong carbodiimide coupling, as well as ballistic electron transport across the electrode–electrolyte interface which are essential for calibrating any sensing device.

### 3.5. Selectivity, Real Sample and Shelf-Life Studies

**Figure 5:**
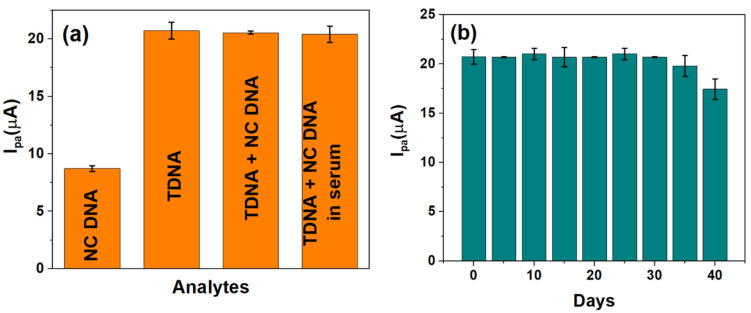
(**a**) Selectivity and real sample analysis and (**b**) shelf-life studies of the developed genosensor over a period of 1 month.

The viability of the developed sensor to selectively detect HPV-16 TDNA upon mixing with non-complimentary DNA (NC DNA) and in serum were evaluated. An amount of 1 pM each of NC DNA and TDNA has been used for performing the selectivity studies, and the corresponding I_pa_ values were observed at the calibrated potential of −220 mV. The current response, as generated by TDNA alone (~20.72 µA), insignificantly differs upon mixing with the NC DNA sequence (~20.56 µA), while the NC DNA yields an I_pa_ ~ 8.7 µA as shown in Figure 5a. Furthermore, human serum samples were spiked with 1 pM HPV-16 TDNA and were also found to exhibit a similar response as to that obtained in buffered redox electrolyte alone, which is in accordance with the calibration in Figure 4b. 

The stability of the developed DNA biosensor was also monitored for 40 days as shown in Figure 5b. It can be seen that the value of I_pa_, at 1 pM HPV-16 TDNA, obtained over 35 days is ~20.84 ± 0.03 µA following which a drastic reduction in I_pa_ was observed; thereby indicating the stability and reproducibility of the sensor over 35 days (~1 month).

## 4. Conclusions

This study reports on the development of a flexible electrochemical DNA biosensor for the detection of HPV-16, which can be used for monitoring the presence of cervical cancer among the women populace, especially in developing nations. The sensor was fabricated using novel CSPE/rGO/DNA bio-nanohybrids, which possess a significant number of carboxyl groups for the efficient anchoring of HPV-16 PDNA. The sensor exhibited excellent linear nature within TDNA concentrations of 1 pM to 1 μM with LoD of ~2 pM, as per differential pulse voltametric calibration achieved at −220 mV. The developed sensor was found to be selective solely to HPV-16 target DNA while exhibiting insignificant deviations in spiked serum samples, along with a shelf life and response time of 1 month and ~15 s, respectively. The flexible nature of the developed sensing platform is considered to lower device expenses and cost per test, which will be of significant aid to patients suffering from HPV infections; eventually leading to faster doctor-patient interactions and positive outcomes. The developed sensing platform can be potentially modified into a multiplexed platform for the monitoring of cervical and breast cancers. This would eventually lead to the development of a packaged platform for the point-of-care monitoring of the two most fatal women-specific cancers—a technology which is currently unavailable in the market.

## Figures and Tables

**Table 1:** Comparison of sensing parameters of the developed HPV-16 biosensor with few reported ones. (PANI: polyaniline; ITO: indium tin oxide; SPGE: screen-printed gold electrode; CSPE: carbon screen-printed electrodes, rGO: reduced graphene oxide).

S. No	Surface Modifier	Linear Range	Limit of Detection (LoD)	Reference
1	Graphene/PANI	10–200 nM	2.3 nM	[39]
2	DNA-modified ITO	0.01–100 nM	3.22 pM	[40]
3	DNA-modified SPGE	5 to 20 nM.	2.39 nM	[41]
4	DNA-modified gold	1 nM–1 μM	0.38 nM	[42]
5	CSPE/rGO/DNA	1 pM–1 µM	2 pM	Present work

## Data Availability

The data presented in this study are available on request from the corresponding author.
